# Why whole body gestational donation must be rejected: a response to Smajdor

**DOI:** 10.1007/s11017-023-09633-3

**Published:** 2023-07-07

**Authors:** Aníbal M. Astobiza, Íñigo de Miguel Beriain

**Affiliations:** Universidad del País Vasco/Euskal Herriko Unibertsitatea, Leioa, Spain

**Keywords:** Whole body gestational donation, Brain death, Surrogacy, Procreation, Morality

## Abstract

Anna Smajdor’s proposal of whole body gestational donation (WBGD) states that female patients diagnosed as brain-dead should be considered for use as gestational donors. In this response, Smajdor’s proposal is rejected on four different accounts: (a) the debated acceptability of surrogacy despite women's autonomy, (b) the harm to dead women ´s interests, (c) the interests of the descendants, and (d) the symbolic value of the body and interests of relatives. The first part argues that WBGD rests on a particular conception of the instrumentalization of bodies that cannot be circumvented simply by the patient’s consent and relinquished autonomy. The second part argues the importance of avoiding any harm to dead women’s interests. The third part identifies the importance of the interest of the foetus in the light of Procreative-Beneficence principle that Smajdor overlooks. And finally, the fourth part considers the symbolic value of the human body and the interest of relatives. The main goal of this commentary is not to show that WBGD cannot be implemented; rather, it is to show that there are not any good arguments in favour of doing so.

## Introduction

In a recent paper, Anna Smajdor [1] discusses the concept of whole body gestational donation (WBGD). She proposes that women who are diagnosed as brain-dead could be used as an alternative to current gestational surrogacy practices.

The concept is not originally hers. Paul Gerber, a bioethicist at Queensland State University, suggested the idea for the first time at an Australian congress in 1988 [2], and the concept was revived in an essay written by Israeli physician Rosalie Ber in this very same journal in 2000 [3]. However, Smajdor introduces some interesting novelties to this framework. Indeed, contrary to both authors, Anna Smajdor considers the use of dead people’s bodies, instead of people in permanent vegetative state (PVS).^1^ This turn seemingly brings some ethical and legal advantages: the fact that dead people cannot suffer and/or their lack of fundamental rights such as autonomy surmounts some of the main issues that Gerber and Ber’s proposals had to face.

At first, the idea of WBGD could be seen as a potential remedy for the scarcity of gestational carriers (often referred to as surrogates) and the high expense of surrogacy. Brain-dead women who have previously given their consent could be used as gestational carriers, making use of otherwise wasted resources. Other people's wishes (e.g., people who want to become parents but cannot) could be fulfilled as a result, allowing for the use of resources that might otherwise go to waste.

Thus, one can hardly neglect that her proposal suggests some relevant advantages. Using the bodies of brain-dead patients who consented to surrogacy before they passed away would, in our opinion, achieve the goal of satisfying the wishes of those who want to become parents through surrogacy without causing harm to alive surrogate mothers. Nonetheless, there are also compelling reasons to reject Smajdor’s proposal.

First, consider the cultural mores and taboos of multiple societies all around the world that prohibit the abandonment of the sacredness of the body—even after death—should they accept this practice? On the other hand, Smajdor’s argument could be seen as objectifying women's reproductive functions and commodifying their reproductive capacity, even if they are dead or previously consented. It could also be seen as sending an implicit message or reinforcement to deeply entrenched assumptions and prejudices against women and the practice of paternalism. Furthermore, there are reasons related to the foetus and the persons they will become that play against WBGD.

In this article, we analyze all these issues carefully, weighing the pros and cons of Smajdor’s proposal. To do so, we focus first on the surrogate woman’s autonomy and consent issues. Then we focus on her interest and the harm the WBGD might cause to her and her child. Finally, we consider the interest of the unborn child and the person to whom it will give rise. We end with some conclusions on the morality of the proposal.

## The debated acceptability of surrogacy despite women's autonomy

### Autonomy and consent

One of the main virtues of Smajdor’s proposal is that, if implemented, it would probably help to avoid some of the worst consequences of surrogacy. In traditional surrogacy, the surrogate mother has a genetic connection to the child. Due to the emotional and psychological effects of carrying and giving birth to a child who is genetically related to her but not her own, this can raise complex issues regarding the surrogate mother´s autonomy, consent, and legal relation to the child.^2^

Concerns exist regarding the surrogate’s ability to give their full consent to the process, especially when they are financially vulnerable or could be forced into it. The surrogate mother's autonomy may also be constrained during the pregnancy because she will have to alter her way of living and the decisions she makes about the pregnancy according to the intended parents’ preferences. Accordingly, Smajdor argues that with WBGD, the scenario of surrogacy would definitely improve, since the problems with consent and limitation of autonomy would be solved. The question is whether this is really the case.

Let us start by considering autonomy. The appeal to a lesser restriction on the autonomy of the pregnant woman is difficult to refute. The freedom of a person who is declared neurologically dead and accepts WBGD is not the same as that of a living person, of course. In this sense, it is true that WBGD affects the autonomy of the brain-dead surrogate mother less than a living mother in traditional surrogacy. However, consent issues are more complex. As commonly known, consent allows for protection from unwanted medical procedures or treatments and is a crucial component of medical ethics and the law.

Consent is the manifest expression of autonomy and—in the context of WBGD—is viewed as a protection to ensure that people are aware of the process and are actively choosing to participate. According to Smajdor, the fact that the brain-dead surrogate mother gave consent to surrogacy before death erases all possible issues connected to consent after death.

This is far from acceptable in our opinion. How does the fact of death change the need for consent to be truly free? Is consent given by a woman who only agrees to surrogacy for economic reasons any freer just because the object of the contract takes place after her death? This does not seem to make much sense. On the other hand, her consent is not the only type of consent that might apply.

### The role of potential harm and exploitation in assessing autonomy

While it is true that brain-dead women do not experience emotional and physical harms associated with pregnancy, one must consider the potential impact on the unborn child, the deceased woman´s relatives, and the medical professionals involved in the process. Even if the risks to the pregnant woman are mitigated (because after brain-death she suffers no emotional or physical harms), there are still potential concerns related to exploitation and autonomy. In the WBGD context, the brain-dead woman’s inability to provide informed consent or express her autonomy remains a significant concern. Without the capacity to actively participate in the decision-making process, the potential for exploitation or violation of the woman’s rights still exists. Furthermore, WBGD may expose the unborn child to additional risks during the gestational process, given the unique medical circumstances. In addition, the relatives of the deceased woman may experience emotional distress and face potential stigmatization or societal pressure. All these factors contribute to concerns of undue influence and exploitation, even if the brain-dead woman herself does not experience emotional or physical harms.

One might think, and we are grateful to an anonymous reviewer for pointing this argument out to us, that to the extent that a woman is declared neurologically dead, the emotional and physical harms are mitigated and therefore undue influence is mitigated. Yes, but it is not enough. And if this is so, namely that there is still a possibility of strong undue influence pressure in the context of WBGD, one might ask—would this not lead to banning “normal surrogacy” as well?

First of all, we must distinguish between the two types of surrogacy: traditional surrogacy and gestational surrogacy. In traditional surrogacy, the surrogate mother uses her own egg and is artificially inseminated with the sperm of the intended father or a sperm donor. The surrogate is genetically related to the child she carries. Traditional surrogacy is less common than gestational surrogacy, partly due to the legal and emotional complexities associated with the genetic relationship between the surrogate and the child.

In gestational surrogacy, the surrogate mother is not genetically related to the child she carries. The intended mother, an egg donor, or a donated embryo provides the egg, which is fertilized in vitro with the sperm of the intended father or a sperm donor. The resulting embryo is then transferred to the surrogate's uterus. Gestational surrogacy is more common than traditional surrogacy and is often preferred because it eliminates the genetic link between the surrogate and the child.

However, both types of surrogacy can be further classified based on the relationship between the surrogate and the intended parents: altruistic surrogacy and commercial surrogacy. We believe that classifying surrogacy in commercial terms would not mitigate the potential for undue influence at all.

If by “normal surrogacy” one means traditional or gestational surrogacy within an altruistic relationship between surrogate and intended parents, and if the parties involved in a surrogacy arrangement are able to provide informed consent, protect the rights and welfare of the surrogate (and of course of the future child), and ensure that no exploitation occurs, then normal surrogacy—understood as altruistic and *not* commercial—may be ethically justifiable based on the principles of autonomy, beneficence (promoting good), and justice (fairness in distribution of benefits and burdens).

### Family-centred autonomy

In contrast, WBGD, even if one accepts that there is no undue influence, is still ethically dubious according to a *family-centered ethics* approach. Some European studies have examined the role of the family and the assessment of patient autonomy in organ donation [5]. What they found is that there are distinct ways of valuing autonomy and family relationships across European regions, such as the authority of the family to overrule the deceased patient’s last wishes. Other surveys indicate that more than 70% of people surveyed felt that family members should have a role in decision-making for their deceased relatives [6], and that it is not clear what to do when patients and family members do not agree. The question is—what role does autonomy play in patient- and family-centered care? If one considers this role as crucial, then the women’s consent should not be considered as the only applicable criterion to solve all consent issues.

In a word, involving the family in the decision-making process related to WBGD is a crucial component of family-centered ethics. Our argument favours a family-centred ethic as a party with a strong counterbalance to a woman who decides to donate her body after her death for an apparently voluntary and free pregnancy. This is because the woman who apparently freely decides to participate in a WBGD practice is only the partial perspective of a multitude of parties involved. The woman's perspective may be tainted by undue exploitation, and adopting a family-centred ethic in the context of WBGD is essential to protect the interests of the deceased woman's relatives as well as the foetus.

### The patient-physician relationship

Finally, it is important to remember that the physician–patient connection is a fiduciary relationship in which the physician is required to act in the best interests of the patient while respecting the patient’s autonomy. If the explicitly expressed desires of an autonomous patient are not in alignment with the physician's, it becomes the physician's responsibility to honor the patient's choice. Furthermore, if the patient has asked to keep their personal preferences concealed, the physician must respect this and keep the patient's wishes confidential. But the role of the family still may have a strong legal mandate in some countries [7].

In the context of WBGD, the physician–patient relationship becomes more complex due to the unique circumstances surrounding the brain-dead surrogate mother and the intended parents. The physician has an ethical obligation to uphold the bioethical principles of beneficence, non-maleficence, autonomy, and justice. In the case of WBGD, the surrogate mother is brain-dead, which raises questions regarding her autonomy and ability to provide informed consent as noted above.

The physician must also consider the potential harms and benefits of WBGD for the surrogate, the intended parents, and the unborn child. These may include how to maintain a body declared neurologically dead throughout a complicated gestational process, the potential for exploitation, and the challenges of determining the best interests of the unborn child.

Effective communication is essential in any physician–patient relationship, but in the case of WBGD it becomes even more necessary. With the intended parents, the surrogate's family, and any other necessary healthcare professionals participating in the surrogate's care, the doctor must always be honest and open. This includes going over any potential hazards, advantages, and alternatives as well as addressing any worries or queries that might arise.

WBGD's complex decision-making process is made more difficult by the surrogate mother's inability to take part in conversations or offer suggestions. The physician–patient relationship in the context of WBGD is therefore multifaceted and requires a thoughtful approach that considers the relevant ethical, legal, and practical implications (as this article is attempting to elucidate).

## The possible harm to the dead woman’s interests

### Brain-dead women’s interests

According to Smajdor, one of the best consequences of using brain-dead women’s bodies is that there is no harm or wrong to these patients. However, some argue that this is not the case. Michael Nair-Collins [8] distinguishes between welfare, experiential, and investment interests in order to analyze the implications of organ procurement from brain-dead donors. Welfare interests refer to the basic needs that are necessary for living well, such as adequate nourishment and hydration, shelter and security, rest, financial stability, etc. Experiential interests involve preferences or desires related to experiences of pleasure or pain in life. These can range from relatively trivial matters (e.g., what food one likes) up to more important decisions about how one lives one’s life (e.g., career choices). Investment interests constitute those things in which an individual has some preference or investment; these include long-term projects that give a person´s life sense and meaning, as well as philosophical/religious commitments regarding the nature of a good death.

Nair-Collins argues that brain-dead patients can be, and many are, harmed or wronged by organ procurement as currently practiced. Other authors [9–11], make similar arguments from different perspectives. This is because the current practice of procuring organs from these individuals does not take into account their welfare interests (e.g., adequate nourishment and hydration), experiential interests (e.g., preferences regarding experiences in life) or investment interests (long-term projects giving a person's life meaning including what to do with their body when they die). In accord with Nair-Collins, we suggest that women declared neurologically dead can have surviving interests that reflect their prior wishes and values; and that these interests can be harmed or wronged by WBGD.

### The importance of investment interests

Investment interests are important because they reflect what is meaningful for individuals, even if their cognitive abilities have been impaired due to illness or injury. As such, organ procurement from brain-dead patients without taking into consideration any surviving investment interest may constitute harm against them, since it ignores their wishes about how they would like to live out the remainder of their lives and what to do with their bodies when they die.

The interconnectedness of interests that Nair-Collins talks about can be categorized under the umbrella term of *moral interests*. This term captures the welfare, experiential, and investment interests discussed by Nair-Collins and their interconnectedness. Moral interests capture the idea that these interests (i.e., welfare, experiential and/or investment) are relevant for the moral identity and treatment of the person, regardless of their physical or mental condition.

It may be the case that one or more of these interests are not present, but given their interconnectedness, the fact that one of them is not literally present does not mean they are not virtually present as moral interests. In the case of a person declared brain-dead who apparently consents to a WBGD, while her experiential or welfare interests may not be present, there are still surviving investment interests.

Even if one grants that brain-dead women only have investment interests, it does not follow that one can avoid harming them by following their “known” preferences. This is because preferences are not always reliable indicators of interests, and they may change over time or in different circumstances. For instance, a woman who expressed a preference for organ donation before becoming brain-dead may have changed her mind if she knew the details of the organ procurement process or if she had a chance to reconsider her values and goals. Therefore, one cannot assume that following the preferences of brain-dead women is sufficient to protect their interests and avoid harming them.

In sum, since it is ethically paramount to balance a patient’s welfare, experiential, and investment interests—or rather moral interests—WBGD is not guaranteed to be an ethically sound practice. One must not forget the interests of the donor, in this case, a patient declared neurologically dead. To harm the interests of the brain-dead pregnant woman is to go against the principle that we call the *Unrealized Experience Principle*.^3^ The principle describes rights or interests not enjoyed by a person.

### The unrealized experience principle

In WBGD, once the brain-dead woman’s body is used to gestate the child of another person, she will no longer have the opportunity to exercise her interests, whatever they might be (e.g., to know the newborn). The rights of surrogates in regard to knowing the baby can vary depending on the laws and regulations of the country or state where the surrogacy arrangement takes place. In some jurisdictions, surrogates may have the right to have contact with the baby, either before or after birth, while in others, the surrogate may not have any legal right to know the baby. In some surrogacy arrangements, the intended parents and surrogate may agree to have an open or closed surrogacy, where the surrogate may or may not have contact with the baby.

This can be outlined in a surrogacy agreement, which is a legally binding document that outlines the rights and responsibilities of all parties involved in the surrogacy process. In the case of WBGD, these interests—in the form of rights—cannot be enjoyed by the person who is declared neurologically dead and who donates her body for gestation. It is important to note that even in cases where surrogates do not have a legal right to know the baby, they may still have an emotional bond with the child and may feel a sense of loss after birth. It is clear that a person declared neurologically dead cannot have this emotional bond, and that is why, in our opinion, there is an overriding interest of this person that is not legitimately satisfied. The *Unrealized Experience Principle* holds that an individual has a right or interest in something only if they are able to enjoy or benefit from it.

If a person is unable to enjoy or benefit from a right or interest, then they are said to not have that right or interest. Neurologically deceased people who donate their entire body for gestation do not enjoy or see the satisfaction of their interests. In the context of WBGD, the *Unrealized Experience Principle* holds that a person who has been neurologically declared dead cannot enjoy or benefit from the rights or interests associated with gestational donation. This means that the gestational donor who is neurologically declared dead has no right or interest in the outcome of the pregnancy or the welfare of the foetus, as they are unable to enjoy or benefit from these things. The *Unrealized Experience Principle* may be used to justify restrictions—or an outright rejection—on the use of WBGD, as it is often seen as unethical to use a person’s body in this manner if they are unable to enjoy or benefit from the rights and interests associated with pregnancy. Note, however that this principle does not require one to admit that the brain-dead woman can continue to have experiences. The costs (harm) incurred during the WBGD procedure and the interests (of which the woman declared dead is deprived) for subjecting her to the WBGD procedure are sufficient to grant the principle valid (as the principle is related to future costs—experienced or not). It is possible then, to cite this principle toward the possibility that, should the brain-dead woman had known the hypothetical and experimental nature of the WBGD procedure, she would have changed her decision to donate her body. This is only understandable by validating her unexercised interests (as this principle seeks to do). Thus, they should be taken into account.

We have described the *Unrealized Experience Principle* as one argument with sufficient justification against the WBGD proposal. The *Unrealized Experience Principle* has implications for the interests of the foetus in the context of WBGD. If a gestational donor is neurologically declared dead and therefore unable to enjoy or benefit from the rights and interests associated with pregnancy, it may be argued that the foetus also lacks these rights and interests, depending on how one views the rights of the mother’s body in relation (over or subordinate) to the rights of the foetal body.

## The interests of the foetus and descendants

### The non-identity argument

In addition to the ethical and societal reasons obstacles discussed above (i.e., welfare, experiential and investment interests, or rather moral interests), there are also compelling arguments against WBGD that consider the interest of the foetus. The interests of the foetus should be protected, regardless of the status of the gestational donor. The foetus has an interest in its own life and health, and this interest should be respected and protected. Moreover, protecting the interest of the foetus means also protecting the interest of the person into which the foetus will become if the developmental process is completed.

This, however, does not directly mean that bringing a person into the world through WBGD is unethical. Indeed, the non-identity argument [12] makes it more likely that WBGD is not hurting the person. This is because, unless one thinks that the person's strange origins will lead to a life that is not worth living, one cannot think that the WBGD is hurting the person.

Indeed, the non-identity principle in its classical formulation explains well why doing something wrong is not the same as causing harm to a specific person. If one considers that the lives of people born through WBGD are reasonably happy, despite the considerable handicaps of the way they were conceived and brought into the world, then none of them could claim that harm has been done to them.^4^

### The procreative-beneficence principle

Thus, if there is anything unethical in terms of how society decides to have descendants, it must be linked to a different line of argumentation. Let us focus now in the so-called *Procreative-Beneficence Principle* proposed by philosopher Julian Savulescu [13]. This principle states that parents or single parents are at least ostensibly required to choose the child out of a range of potential offspring who will be most likely to lead the best life. Does WBGD work well with the *Procreative-Beneficence Principle*?

WBGD is a very experimental or hypothetical technology, and the effects of being able to carry a pregnancy to term within a woman in a brain-dead state—without putting the foetus at risk—are not known.^5^ What we know for sure is that, with a living woman, the foetus and its future is less uncertain. One must also consider, before resorting to WBGD, that there are other alternatives that can be explored, such as adoption, which may not allow for a genetic relationship with the future baby but does not carry the problems we are describing here. For example, WBGD might lead to the exploitation and commodification of women's bodies, as they could be reduced to mere gestational carriers. This could be seen as contrary to the *Procreative-Beneficence Principle*, as it may not be in the best interests of future generations if their mothers are treated as mere commodities. Thus, in the context of WBGD, the use of a deceased woman’s body to carry a foetus to term can be seen as incompatible with the *Procreative-Beneficence Principle* as traditional surrogacy and adoption are more beneficent alternatives given their studied impacts on the development of the child.

To ensure the greatest possible level of wellbeing for their children, parents must make informed decisions regarding having children according to the *Procreative-Beneficence Principle.* WBGD, however, has the potential to compromise the gestational carriers' autonomy and informed consent because they can feel under duress or pressure to carry a pregnancy for someone else, even after death.

WBGD involves novel medical procedures and risks associated with pregnancy and childbirth. While the *Procreative-Beneficence Principle* promotes the pursuit of reproductive technologies to improve the chances of having a healthy child, it does not advocate for exposing children to unnecessary medical risks.

### Abortion and feticide

There is an additional issue that should also be considered in connection with the foetus and its interests. In her paper, Smajdor states that:

…in places where embryonic research is permitted, the law often allows for abortion. Legal grounds for abortion generally include impairments or diseases affecting the foetus. Thus, with very close surveillance, it is reasonable to think that–if foetuses are severely damaged by unexpected factors arising from brain-dead gestation–this need not result in the birth of severely damaged babies. Rather, it could result in the termination of the process at the discretion of the commissioning parents. Abortion, especially late-term abortion, can be traumatic for gestating women both emotionally and physically. However, in the case of WBGD, the gestating woman is already dead and cannot be harmed. Commissioning parents may decide on abortion or selective reduction in accordance with their own wishes, without having to worry about the effects on the gestating donor [1, p. 120].

Her argument, therefore, is very simple: what makes the question of abortion in surrogacy problematic is that the interests or feelings of the surrogate may differ from those of the intended parents. Since a brain-dead person has no interests, it would be perfectly permissible to give the intended parents the right to decide about the future of the foetus.

In our opinion, this argument has an important handicap since it starts from a more than debatable premise. Here, Smajdor is equating the right to abortion with the right to feticide. This is not strange because, when a woman decides to end her pregnancy, the foetus usually dies because the pregnancy cannot continue outside of her body. Conceptually, however, the two rights are different [14]. This can be clearly seen from the moment of fetal viability—at that moment, it is possible to terminate (abort) a pregnancy by causing childbirth without causing the death of the foetus [15].

So, we believe it is a theoretical mistake to think that the right to end a pregnancy includes the right to kill the foetus, as feminist thinkers like J.J. Thomson [16] have pointed out, and as a long-standing tradition continues to point out.

In fact, in the context of a brain-dead woman, it would be logical to think that she has no right to terminate her pregnancy (or any other, of course, since she is dead and dead people have interests, but not rights [17]). To infer from this that this right passes to the intended parents would only be sound if one believes that it covers the right to feticide. But if this were not the case, as many jurisdictions consider feticide a crime, it is obvious that they would have no right in this respect.^6^

Another, of course, is to advocate for terminating the pregnancy out of respect for the dignity of the pregnant woman's body or to prevent the birth of a child with serious disabilities via prenatal euthanasia (which is also illegal in many countries). But none of this has anything to do with Smajdor's argument. As a result, one must conclude that she is incorrect, at least in this specific issue.

## The symbolic value of the human body and the interest of the relatives

There are additional reasons to reject Smajdor’s proposal. First, one must consider the effect that WBGD may have on societal values, such as the symbolic value of purity and sacredness of the body and related norms. Since WBGD entails the exploitation of women's bodies, even though they are no longer living, surrogacy in the case of brain-dead women is morally and ethically reprehensible if one takes into account the social value(s) of the purity of the body and body symbolism, etc.

The purity and sacredness of the body in the context of WBGD cause a much stronger visceral and emotional reaction than in the context of organ donation (which are parts of a body). Understanding the key differences between the two procedures and the particular ethical issues they pose is crucial when pondering why the “purity and sacredness” of the human body applies differently to WBGD than to organ donation.

Organ donation has a well-established purpose of saving or improving the lives of individuals with organ failure. The benefits of organ donation are tangible and widely accepted which has contributed to a greater societal understanding and acceptance of the practice, despite potential concerns about the sacredness and purity of the human body.

Depending on one's cultural, religious, and personal beliefs, one's understanding of organ donation and WBGD may differ greatly. Some people may consider WBGD as a breach of the sanctity of the human body, particularly when it comes to utilising a woman's body for reproductive reasons (i.e., reducing her body to a reproductive asset), while some may see organ donation as a caring and self-sacrificial act that is consistent with their values. The extent of bodily involvement may evoke stronger feelings (e.g., the “yuck effect” [18]) and lead to different ethical considerations (e.g., specific organs or tissues are not the same as the whole body).

In sum, everything lies in the visceral, emotional, and affective reaction that is behind the taboo of the sacredness or purity of the body as a whole, as opposed to some parts of the body, such as organs. However, transplants of some parts of the body also cause the same reaction. For example, many people identify the face as something so personal that they would be reluctant to have the face of another person transplanted onto them.

In addition, some religions, such as Christianity, believe in the resurrection of the body. Accordingly, reconciling WBGD with the belief in the resurrection of the body is a controversial issue. Moreover, a long tradition of liberal feminism also offers arguments against WBGD beyond the sacredness and purity of the body. For example, there are arguments that emphasise bodily autonomy, consent, and the potential emotional, psychological, and medical implications for vulnerable and marginalized groups of people.

On the other hand, there is no compelling reason or “overriding interest” to justify the instrumentalization of the body for the purpose of gestational donation. One of the most important issues to consider is the question of what kind of impact WBGD may have on the grieving process of the brain-dead patient's family, loved ones, and even society at large. In the context of organ donation, this level of consideration is not uncommon [19]. First, because society is well aware of the anguish that relatives may experience if a gift is not accepted and/or the social impact of using bodies against their desires. Second, a rapid donation—in which the organs are extracted and the body is buried or burned and differs from a normal donation that takes months—may cause increased stress on the deceased's family members; this appears to be a very different situation. This should also be balanced against the possible benefits that WBGD involves.

We thank an anonymous reviewer for pointing out the potential inequality of access to this practice of WBGD, which for many is nothing more than a technocratic response that only the most affluent in society will have access to and the poor will not. Or, in worse circumstances, the wealthy will satisfy their desires with this technocratic solution while using the bodies of the non-wealthy, within a neoliberal political context of extraordinary inequality of wealth and political power. Despite the fact that it will be expensive and expend a lot of resources, it will never help the vast majority of people of this world. We agree with this reviewer that this political-economic aspect would be sufficient to reject WBGD without having to use bioethical arguments like the ones we have pointed out here. At the same time, justice is a principle of biomedical ethics and factors in issues such as these.

## Conclusions

The idea of WBGD has been suggested as an alternative to the way that surrogacy is currently done. This idea, advanced by Anna Smajdor, is to employ the bodies of women who have given their consent and are brain-dead as gestational carriers. While there are benefits to this idea, such as the ability to employ resources that would otherwise go to waste and the satisfaction of individuals who desire to become parents, there are also strong arguments against it. As this paper has shown, there is the debated permissibility of surrogacy despite women’s autonomy; the potential harm to the brain-dead women’s interests; the objection under the *Unrealized Experience Principle*; the role of the family and other overriding interests, such as the interests of the foetus in the light of the *Procreative-Beneficence Principle;* and the symbolic value of the body. All of these arguments held together suggest rejecting WBGD on ethical and bioethical grounds. The non-identity argument suggests that creating a person through WBGD is not unethical as long as the resulting life is reasonably happy, but the *Procreative-Beneficence Principle* may not be compatible with this method. On top of that, while a brain-dead gestational donor does not have the full range of moral interests, as we have described above, it is debatable whether the intended parents have the right to terminate the pregnancy or perform selective reduction without considering the interests of the foetus. In this article, we have sought to articulate more concretely all these objections to Smajdor's proposal to facilitate further discussion and reflection on this important debate.

## Footnotes

1 Since Jennett and Plum coined the term in their landmark paper in *The Lancet* more than 50 years ago, the biomedical community and civil society are beginning to express their dissatisfaction with the term because of its pejorative connotations. Several physicians have proposed a neutral term “unresponsive wakefulness syndrome” [4]. We believe this term is more appropriate. So, although Smajdor, or even Ber, refers to PVS, we will use “unresponsive wakefulness syndrome” instead (abbreviated UWS).

2 See the United States legal case, in re: Baby M. We thank an anonymous reviewer for pointing this out.

3 Initially, we called this principle the Non-Enjoyment Principle. But thanks to the suggestion of an anonymous reviewer, “experience” is the proper word to capture the point and shatter assumptions that having interests is equated with enjoyment, which is less true for those suffering oppression. This principle refers to those interests neglected of the dead woman—such as the interest in meeting the future baby—that WBGD renders her incapable of enjoying. Other types of interests that are not enjoyed—or rather experienced—are (just to name a few): the interest of having a respectful treatment after death and not being treated as a means to an end; the interest of not being subject to uncertain, risky, experimental medical interventions or procedures even after death; and the interest of retaining a good memory with those who survive her by not being reduced to a mere incubator; the interest of experiencing motherhood bonding with her baby (even if it is contractually stipulated that no contact with the baby is to be maintained). The list of interests could go on and on.

4 We are grateful to an anonymous reviewer for pointing out to us that “reasonably happy” does not mean someone does not have trauma to heal from (trauma resulting from phenomenon happening to someone outside their control). Accordingly, it may be worth considering the neuro-cognitive and developmental trauma a child gestated this way could experience and have to face throughout their life. That itself—for many—could be considered significant harm (but not enough to justify antinatalism!).

5 All that *is* known are the cases about maternal somatic support (i.e., support for the gestating children in the wombs of patients who have been declared brain-dead).

6 In other words, the intended parents may think they are terminating the pregnancy in a person declared dead when they are really just killing the foetus. Intending parents should not have the right to abortion as it would include the right to kill the foetus, which is extraordinary and unlawful in many jurisdictions.
